# Evaluation of Appropriate Reference Genes for Reverse Transcription-Quantitative PCR Studies in Different Tissues of a Desert Poplar via Comparision of Different Algorithms

**DOI:** 10.3390/ijms160920468

**Published:** 2015-08-28

**Authors:** Hou-Ling Wang, Lan Li, Sha Tang, Chao Yuan, Qianqian Tian, Yanyan Su, Hui-Guang Li, Lin Zhao, Weilun Yin, Rui Zhao, Xinli Xia

**Affiliations:** 1 National Engineering Laboratory for Tree Breeding, College of Biological Sciences and Technology, Beijing Forestry University, Beijing 100083, China; E-Mails: whling@bjfu.edu.cn (H.-L.W.); lilan@bjfu.edu.cn (L.L.); yuanchao@bjfu.edu.cn (C.Y.); tqqetqq@bjfu.edu.cn (Q.T.); yanyansu@bjfu.edu.cn (Y.S.); hg_li@bjfu.edu.cn (H.-G.L.); lynnzhao@bjfu.edu.cn (L.Z.); yinwl@bjfu.edu.cn (W.Y.); 2 The Key Laboratory for Silviculture and Conservation of Ministry Education, College of Forestry, Beijing Forestry University, Beijing 100083, China; 3 Institute of Crop Sciences, Chinese Academy of Agricultural Sciences, Beijing 100081, China; E-Mail: tangsha@caas.cn

**Keywords:** reference genes, salt stress, normalization, reverse transcription-quantitative PCR, *Populus euphratica*

## Abstract

Despite the unshakable status of reverse transcription-quantitative PCR in gene expression analysis, it has certain disadvantages, including that the results are highly dependent on the reference genes selected for data normalization. Since inappropriate endogenous control genes will lead to inaccurate target gene expression profiles, the validation of suitable internal reference genes is essential. Given the increasing interest in functional genes and genomics of *Populus euphratica*, a desert poplar showing extraordinary adaptation to salt stress, we evaluated the expression stability of ten candidate reference genes in *P. euphratica* roots, stems, and leaves under salt stress conditions. We used five algorithms, namely, Δ*C*_t_, NormFinder, geNorm, GrayNorm, and a rank aggregation method (RankAggreg) to identify suitable normalizers. To support the suitability of the identified reference genes and to compare the relative merits of these different algorithms, we analyzed and compared the relative expression levels of nine *P. euphratica* functional genes in different tissues. Our results indicate that a combination of multiple reference genes recommended by GrayNorm algorithm (e.g., a combination of *Actin*, *EF1*α, *GAPDH*, *RP*, *UBQ* in root) should be used instead of a single reference gene. These results are valuable for research of gene identification in different *P. euphratica* tissues.

## 1. Introduction

Gene expression characterization at the steady-state mRNA level is an important step in many plant biological processes. Northern blotting [1,2,3,4], is not an optimal method for gene expression quantification anymore, because it requires a large amount of RNA and is limited to detecting high-abundance transcripts. Therefore, reverse transcription-quantitative polymerase chain reaction (RT-qPCR), which can detect low-abundance mRNAs and exhibits high specificity, sensitivity, and stability [5,6,7], has become one of the most commonly used techniques [8]. However, RT-qPCR has certain disadvantages; for example, reference genes are important to correct the experimental error that occurs during RNA extraction, and cDNA preparation, but the normalized results are highly dependent on the expression stability of the reference genes selected [9]. The use of inappropriate reference genes can result in biased target gene expression profiles; thus, using suitable reference genes for data normalization is a prerequisite for any RT-qPCR study [10]. The expression of an ideal reference gene should not vary between different tissues or cells under investigation, nor in response to any experimental treatment [11]. However, extensive transcriptomic data mining and gene expression analysis studies have reported that the reliability of these endogenous controls can be influenced by various conditions [12,13,14,15,16,17]. Therefore, to obtain accurate and reliable target gene expression profiles, it is important to identify the best reference genes for use in each experimental set-up and verify these in each individual experiment before performing gene expression normalization. In common plant species, including *Arabidopsis*, rice, soybean, and petunia, genes associated with basic cellular processes such as those related to ubiquitin degradation, polyubiquitin, ubiquitin-conjugating enzymes, and ubiquitin ligases (collectively known as *UBQ*), as well elongation factors (EFs) [18,19,20,21], have been identified and used for RT-qPCR [6,12,13,19]. Nevertheless, few reference genes have been identified in forest trees, which dominate much of the terrestrial landscape on earth. Members of the genus *Populus* are used as a model forest species for diverse studies because of their amenability to experimental and genetic manipulation [22,23,24,25]. Studies in *Populus* focus mainly on improving their ability to respond to environmental stress while maintaining their high-speed growth capacity because there is strong demand for their cultivation in highly saline and arid soils in many parts of the world [26,27]. *Populus euphratica* Oliv., which is native to desert regions ranging from western China to North Africa, is the only arboreal species established in the world’s largest shifting-sand desert, the Taklimakan Desert in China [28]. The most significant characteristic of *P. euphratica* is its extraordinary adaptation to salt stress [28,29,30]. This species can survive concentrations of NaCl in nutrient solution up to 450 mM while maintaining higher growth and photosynthetic rates than other poplar species [31,32,33]. Previous studies of *P. euphratica* have mainly focused on analyses of salt-responsive genes [26,30,34,35], miRNAs [36,37], or salt-related transcriptome sequencing [26,38,39]. In these studies, the genes up-regulated or down-regulated by salt stress are important and should be identified using validated reference genes by RT-qPCR.

Here, we evaluated the expression stability in *P. euphratica* leaves, stems, and roots under salt stress of ten previously used reference genes [40,41], which are 60S ribosomal protein L35 (*60S*), *Actin*, elongation factor-1 α (*EF1*α), eukaryotic initiation factor 5A (*eIF-5A*), glyceraldehyde-3-phosphate dehydrogenase (*GAPDH*), glucosidase II α-subunit (*GII*α), histone superfamily protein H3 (*HIS*), ribosomal L27e protein family (*RP*), tubulin β chain (*TUB*) and ubiquitin family 6 (*UBQ*). Five different methods were used for identification of suitable reference genes and the expression normalized by the reference genes selected by these methods was investigated for nine *P. euphratica* genes; namely, *PeCOBL4* (COBRA-like extracellular glycosyl-phosphatidyl inositol-anchored protein family 4), *PeFLA12-1*, *PeFLA12-2*, *PeFLA12-3* and *PeFLA12-4* (FASCICLIN-like arabinogalactan-protein 12), *PeHKT1* (high-affinity K^+^ transporter 1), *PeKUP3* (K^+^ uptake transporter 3), *PeNhaD1* (NhaD-type Na^+^/H^+^ antiporter 1), and *PeNHX2* (Na^+^/H^+^ exchanger 2). The analyses show that the different reference genes identified by different methods had a variable impact on gene of interest data, and that a reliable set of reference genes should be selected based on the highest possible accuracy of RT-qPCR results. This approach can be applied to future studies of gene expression profiles in *P. euphratica*.

## 2. Results

### 2.1. RNA Quality and PCR Specificity

**Figure 1:**
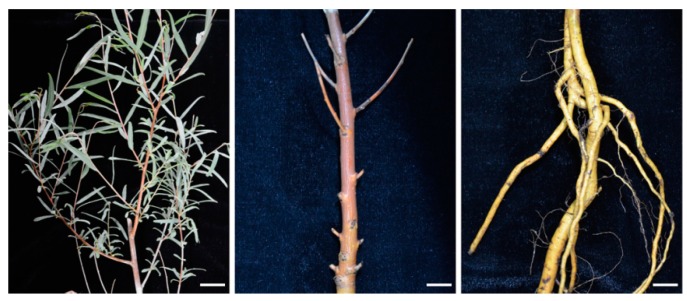
Leaves, stems and roots used for RNA extraction. Mature leaves, stem epidermis and healthy roots were collected from salt-stressed *P. euphratica* saplings. Bars = 2 cm.

This study was performed in accordance with the minimum information for publication of reverse transcription-quantitative PCR experiments (MIQE)-guidelines (Table S1). *P. euphratica* saplings (Figure S1) were exposed to salt stress for 0, 1, 3, 6, 9 and 12 h and then leaves, stems and roots were sampled for RNA extraction (Figure 1). The time-point “0” was used as a reference point, and sampling at further time-points represents a combination of salt-induced changes in gene expression as well as diurnal changes. Based on agarose gel electrophoresis, the intensity of the *25S* rRNA band was nearly twice that of *18S* rRNA band, and no genomic DNA was observed (Figure S2). Using a spectrophotometer, the OD_260_/OD_280_ ratios of total RNA were between 1.8 and 2.0, and the OD_260_/OD_230_ ratios were greater than 1.5. Using an Agilent 2100 Bioanalyzer (Agilent Technologies, Santa Clara, CA, USA) for total RNA integrity detection, no evidence of degradation was observed. After cDNA synthesis, qPCR using the primers listed in Table 1 was performed, and to verify the specificity of these primers, the amplified products were analyzed using 2% agarose gel electrophoresis and only one band of the expected size was observed in each experiment. Meanwhile, also the presence of a single peak in the melting curve was observed (Figure S3).

**Table 1:** Gene names, Gene IDs, Primer sequences, and PCR efficiencies of the ten *P. euphratica* candidate reference genes and nine functional genes for RT-qPCR analysis. ^e^

Gene Name	Phytozome v9.1 GI (*P. trichocarpa*)	Primer Sequences (5′–3′, Forward/Reverse)	PCR Efficiency
*60S* ^a^	Potri.007G093700	AGGTGAACTCTTGATGCTTCGTCTT/CTTCTCTTCCATTGCCTGTCCAACT	1.972
*Actin* ^a^	Potri.006G192700	AAGATTCCGTTGTCCAGAGGTCCT/GAACATAGTAGAGCCACCACTGAGAAC	2.027
*EF1*α ^a^	Potri.006G130900	TCCGTCTTCCACTTCAGGATGTCT/GTCACGACCATACCAGGCTTCAG	1.896
*eIF-5A* ^a^	Potri.018G107300	TCGGACGAGGAGCACCACTT/TGCAAGGACGGTTCTTGATGACTAT	1.965
*GAPDH* ^a^	Potri.010G055400	ATGAAGGACTGGAGAGGTGGAAGG/CACAGTAGGAACACGGAAGGACATT	1.904
*GII*α ^a^	DQ388455.1 ^d^	CTCTCATTGAGCCGGCAAAT/CCCCCCTTCAAGCATAAGG	2.009
*HIS* ^a^	Potri.005G072300	ACTGCTCGTAAGTCTACTGGAGG/GCGGTAACGGTGAGGCTTCTTC	1.968
*RP* ^a^	Potri.001G342500	GTTACACGCTGGATGTGGACTTG/AACCACCTGTTCTTGCCTGTCTT	1.935
*TUB* ^a^	Potri.003G126800	GGAGGTGGAACTGGATCAGGAATG/GGCATTGTAAGGCTCAACCACTGT	1.914
*UBQ* ^a^	Potri.014G115100	AGACCTACACCAAGCCCAAGAAGAT/CCAGCACCGCACTCAGCATTAG	2.097
*PeCOBL4* ^b^	Potri.004G117200	GCACTTACTCACAATTCATGGCAAG/TTGGCAACCGCACGCACAA	1.887
*PeFLA12-1* ^b^	Potri.009G012100	TTATCTTGTTATTCTGGCTCCTCTTCCTC/TGTCTGAGTCGCTGCTGGTG	1.944
*PeFLA12-2* ^b^	Potri.009G012200	TGGAATAACAATCCTGGCACCAACTG/GCTCAGTCTTGTCTTCATCGCTTAG	2.031
*PeFLA12-3* ^b^	Potri.012G015000	ACTTACCAATACAAGCGTATCGGCAAT/TGGAGCAGGAGCAAGAGGTTTAGG	2.074
*PeFLA12-4* ^b^	Potri.004G210600	ACTTCACTGTCTTCGTCCGCCTAAT/CCAACTCAGTCTTGTCTTCATCGCTT	2.049
*PeHKT1* ^c^	Potri.018G132200	TCTTGGTGCTCTTCGTGGTTATGATG/CAAAGATGGCTAAGGTAGATAAAGGTGAG	2.054
*PeKUP3* ^c^	Potri.014G144900	CAATCAACAAGCAGCCGATGAGG/GGAGTAAGCACGCCATCACCTATG	1.978
*PeNhaD1* ^c^	JX981308 ^d^	GGACTCTTCTTTGGGTGGTTGGTTT/GCTTGCGGTATTCTGATGGAGGTAC	1.954
*PeNHX2* ^c^	Potri.014G134900	GACACGGTGGATTATCTAGGCTTGG/CTCGAGGTGATGTGTGAGAGGTC	2.062

^a^ Reference gene candidates [40,41]; ^b^ [32]; ^c^ [26] Genes used to support the suitability of the identified reference genes; ^d^ GenBank accession number; ^e^ The RT-qPCR efficiency was determined by standard curve method and then confirmed by LinReg PCR program.

### 2.2. C_q_ Value Distribution and Expression Profile of the Ten Reference Genes

**Figure 2:**
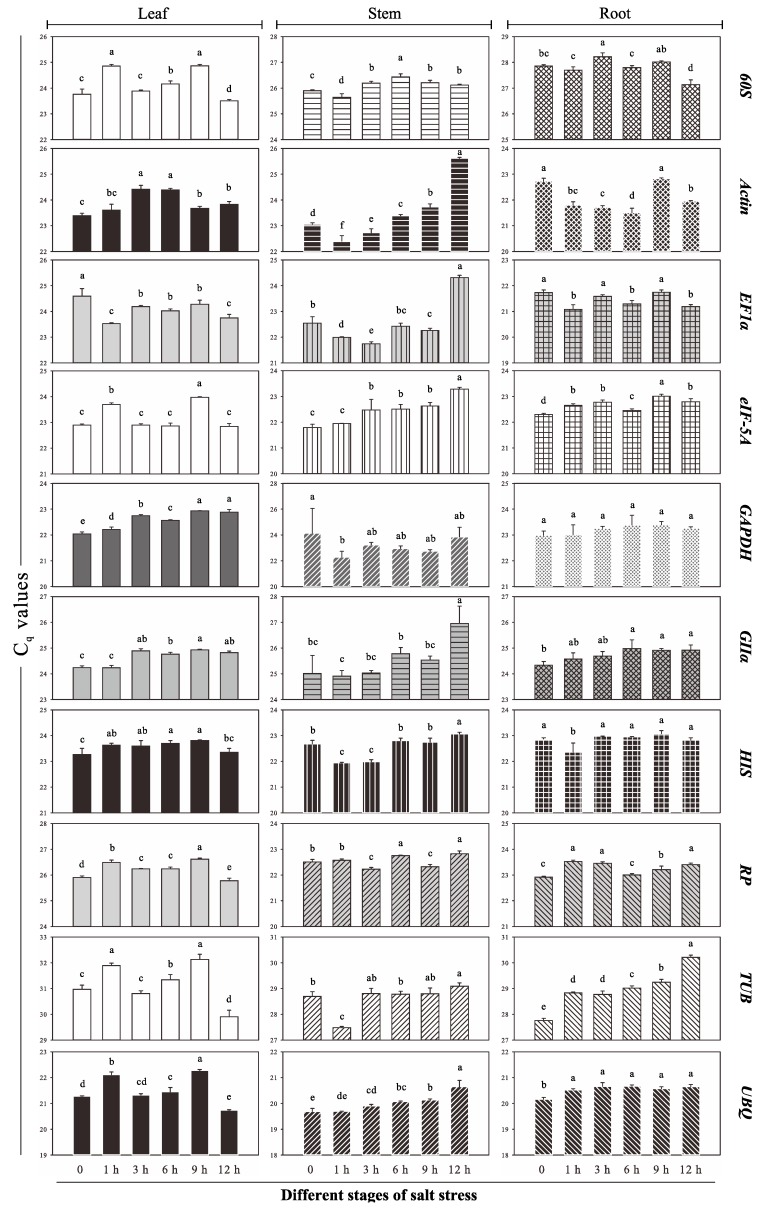
Quantification cycle (*C*_q_) values for candidate reference genes in all *P. euphratica* samples. Letters indicate significant differences at *p* < 0.05 using least significant difference (LSD) test (Average ± SD).

The detailed information of all the genes, primers and amplicons can be seen in Table 1 and Table S2. All RT-qPCR assays were performed on biological triplicate samples to determine the expression rates of the selected genes. Firstly, for each candidate reference gene the average *C*_q_ values of these triplicates (for each condition and tissue) were visually presented using a histogram. This allows the expression levels of the genes to be easily compared (Figure 2 and Figure S4). One-way analysis of variance results were used to show differences in measured non-normalized expression. The *C*_q_ values ranged from 19.61 to 32.36 in root, stem, and leaf samples. *UBQ* showed the highest expression level irrespective of whether all tissues or just one tissue were considered. The *C*_q_ values of most of the genes ranged from 22 to 26 except *TUB* and *UBQ*. *TUB* showed the lowest expression level with a mean *C*_q_ value of 29.58 while *UBQ* overall showed the highest expression. When looking at these non-normalized data, the observed differences in measured expression within one gene under salt stress are due to both technical and biological variation. Normalization of target gene expression with reference genes will eliminate technical variation, but also erroneously impose any biological variation in reference gene expression. Therefore the expression reference genes should be minimally influenced by the salt treatment. However, these results demonstrate that the reference genes were not perfectly stable under salt stress. Indeed, considering the gene expression variation under salt stress in each tissue type, *HIS* in leaf (0.54 cycles), *RP* in stem (0.60 cycles), and *UBQ* in root (0.51 cycles) showed the lowest variance, while *TUB* in leaf (2.23 cycles), *GII*α in stem (3.33 cycles), and *TUB* in root (2.46 cycles) showed the highest variance. This different variance in measured expression for these ten reference genes under salt stress cannot only be due to technical variation but shows that there is a biological component in reference gene variation. It is necessary to identify reference genes that have a minimal biological variation component prior to performing target gene expression studies.

### 2.3. Statistical and Bioinformatical Analyses of Gene Expression Stability Using ΔC_t_, NormFinder, geNorm, RankAggreg and GrayNorm

Given the indications for variations in expression, it was important to evaluate the stability of the ten candidate reference genes. Data from leaves, stems, or roots was analyzed separately. Many approaches exist, and to compare these, statistical and bioinformatical analyses of the data were performed according to the flowchart shown in Figure S5.

#### 2.3.1. Δ*C*_t_ Algorithm

**Figure 3:**
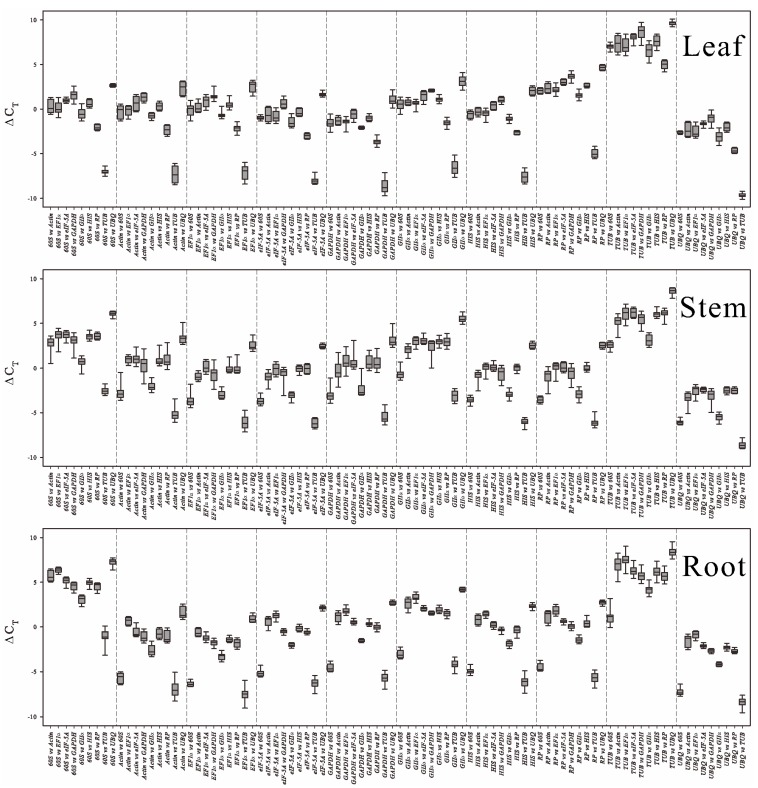
The Δ*C*_t_ method of reference gene selection exhibited by box-whisker plots in all *P. euphratica* tissues. Box-whisker plots show the *C*_t_ variation of the samples between different reference genes. The longer the box the larger the deviation in the Δ*C*_t_ values of the two reference genes, indicating that one or both genes were variable in the test samples. Similarly, the shorter box means smaller deviation, indicating that both genes showed relatively stable expression or were co-regulated with each other.

In the Δ*C*_t_ algorithm [42], if the Δ*C*_t_ value between two genes (the *C*_q_ value of one gene minus the *C*_q_ value of a second) remains constant when analyzed in different cDNA samples, then either both genes are stably expressed among the samples or they are co-regulated. Meanwhile, if the Δ*C*_t_ value fluctuates, then one or both genes are variably expressed. By introducing a third, fourth or fifth gene into the comparisons, more information is available on pairs showing less variability, and they can then be ranked or discarded [42]. Whether the distribution of Δ*C*_t_ values between one gene and the other nine genes was discrete or concentrated was visually presented using a box-plot. Figure 3 and Table 2 use this Δ*C*_t_ approach to reference gene selection among different tissues under salt stress.

The expression stabilities were evaluated via mean standard deviation (mSD), and the mSD values were calculated through Δ*C*_t_ values between one gene and the other nine genes. The smaller mSD value of one candidate reference gene indicates more stable expression. By calculating each gene’s mSD of the Δ*C*_t_ values, we obtained the final rankings; the most stable gene showed the top ranking (Table 2).

**Table 2:** Candidate reference genes ranked according to their expression stab. (stability, Mean StdDev values) based on Δ*C*_t_ method.

Rank (Position)	Leaf		Stem		Root	
	Gene	Stab	Gene	Stab	Gene	Stab
1	*RP*	0.384	*UBQ*	0.517	*UBQ*	0.366
2	*HIS*	0.407	*HIS*	0.547	*eIF-5A*	0.369
3	*eIF-5A*	0.464	*eIF-5A*	0.577	*GAPDH*	0.385
4	*UBQ*	0.465	*RP*	0.612	*GII*α	0.399
5	*60S*	0.472	*60S*	0.621	*HIS*	0.403
6	*GII*α	0.494	*TUB*	0.627	*RP*	0.431
7	*GAPDH*	0.519	*GII*α	0.660	*EF1*α	0.441
8	*Actin*	0.610	*EF1*α	0.689	*60S*	0.503
9	*EF1*α	0.615	*Actin*	0.816	*Actin*	0.636
10	*TUB*	0.655	*GAPDH*	1.012	*TUB*	0.772

**Figure 4:**
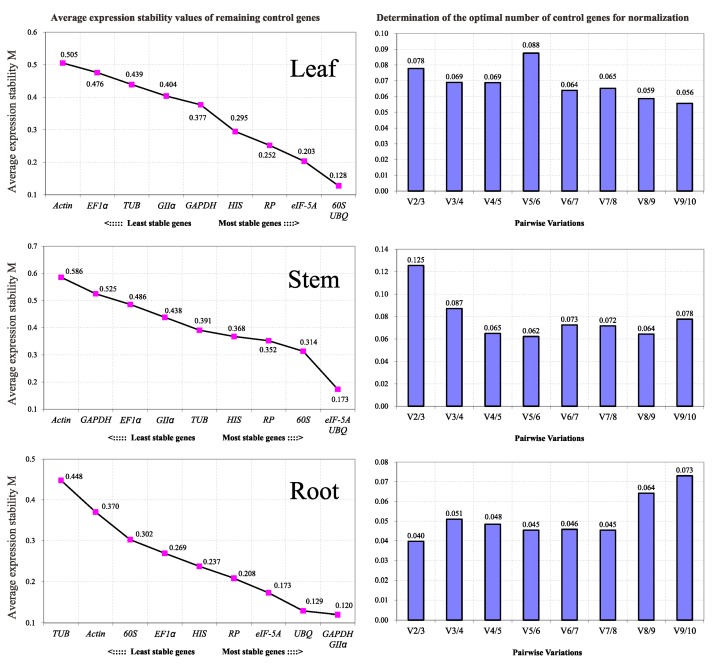
geNorm-based evaluation of candidate reference gene expression stability (the left three line charts) and determination of the optimal number of reference genes for normalization (the right three histograms). The lowest values of the expression stability measure M indicate the most stable expression. The pairwise variation *V_n_*_/*n* + 1_ value is an indication of number of genes to include for normalization. Additional reference genes should be included in the analysis until the *V_n_*_/*n* + 1_ value is below the 0.15 threshold, or until a minimum is reached.

#### 2.3.2. Normfinder

Secondly, the NormFinder tool was used to identify the most suitable reference gene [43]. At least three genes and two samples per group were required for analysis using this algorithm. In our study, ten genes and six samples per group were performed. Based on amplicon efficiency, the *C*_q_ values were log-transformed and used as input [43]. Table 3 indicates that in leaves under stress, *RP* was the most stable gene (stability value = 0.024), while *Actin* was the most unstable gene (stability value = 0.164). *UBQ* and *eIF-5A* were the most stable genes in stem and root, respectively.

**Table 3:** Expression stability values of the ten candidate reference genes calculated using the NormFinder tool.

Rank (Position)	Leaf		Stem		Root	
	Gene	Stab	Gene	Stab	Gene	Stab
1	*RP*	0.024	*UBQ*	0.048	*eIF-5A*	0.031
2	*HIS*	0.031	*HIS*	0.071	*GAPDH*	0.040
3	*eIF-5A*	0.087	*eIF-5A*	0.082	*UBQ*	0.042
4	*60S*	0.098	*TUB*	0.108	*GII*α	0.053
5	*GII*α	0.110	*GII*α	0.118	*HIS*	0.055
6	*UBQ*	0.112	*RP*	0.120	*RP*	0.073
7	*GAPDH*	0.114	*60S*	0.132	*EF1*α	0.078
8	*EF1*α	0.140	*EF1*α	0.132	*60S*	0.116
9	*Actin*	0.155	*Actin*	0.218	*Actin*	0.171
10	*TUB*	0.164	*GAPDH*	0.241	*TUB*	0.206

#### 2.3.3. geNorm

For the geNorm tool, *C*_q_ values were transformed to quantities based on *E*^−Δ*C*q^ (*E* = efficiency), and in each gene among different samples the highest quantities were set to 1 to obtain the input [11]. The average expression stability value, *M*, was calculated to rank the stability of the candidate genes. The genes with the lowest *M* value show the most stable expression. However, it should be noted that the strongly co-regulated genes also show low *M* values. The geNorm algorithm recommended an *M* value below a threshold of 1.5 for a gene to be considered stably expressed. However, a stricter standard using 0.5 as the threshold has also been accepted [20,44]. Based on the ranking results shown in Figure 4, almost all genes showed highly stable expression in leaf and root. According to geNorm analysis, the most optimal reference genes for RT-qPCR normalization were *60S* and *UBQ* in leaf, and *eIF-5A* and *UBQ* in stem, *GAPDH* and *GII*α in root.

Quantification of target gene expression relative to multiple reference genes requires calculation of the normalization factor (*NF*), the geometric mean of the expression of combined reference genes. geNorm software computes *NF* values. To determine the minimum number of reference genes to be used for normalizing the experiment, the pairwise variation (*PV*) of two sequential NFs (*V_n/n +_*_1_) was used to estimate the effect of introducing additional reference genes to the NF [11]. If the PV value for n genes (*V_n_*_/*n* + 1_) is below a threshold of 0.15, *n* can be considered the minimum number of reference genes. Alternatively, *n* + 1 genes can be used when the (*V_n_*_/*n* + 1_)-graph reaches a minimum [45]. In our study, the expression of the ten genes varied greatly in stems, such that V_5/6_ was used in stems. V_2/3_ was used in leaf and root, but it is advisable to use at least three genes to reduce the chance of selecting co-regulated genes. Thus, three reference genes should be used for normalization in leaf and root, and six in stems (*n* + 1 at minimum *V_n_*_/*n* + 1_).

#### 2.3.4. RankAggreg

**Figure 5:**
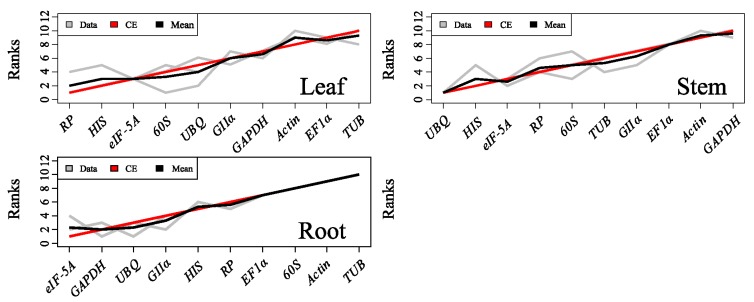
Rank aggregation of ten genes lists using the Monte Carlo algorithm. Visual representation of rank aggregation using RankAggreg with the Monte Carlo algorithm and Spearman footrule distances. The ten candidate reference genes were ordered based on their rank position according to three stability detection methods, Δ*C*_t_, NormFinder and geNorm (gray lines). The mean rank position of each gene is shown in black, while the model computed using the Monte Carlo algorithm is indicated by a red line.

Since the different methods showed different rankings results for each gene, we used the RankAggreg statistical method to create an aggregate order to obtain a final list of genes for each tissue. The rank positions generated using the three statistical approaches were merged, including mSD values of Δ*C*_t_, stability values of NormFinder, and *M* values of geNorm. The results in Figure 5 indicate that the most adequate genes tested for normalization in leaf were *RP*, *HIS*, and *eIF-5A*. In stem, the best three candidate genes were *UBQ*, *eIF-5A*, and *HIS*. In addition, *eIF-5A*, *GAPDH* and *UBQ* were top-ranked in root.

#### 2.3.5. GrayNorm

GrayNorm is a recently published algorithm that finds a combination of reference genes that have the smallest possible deviation from the non-normalized data, thus are carrying the least biological variation while they are used for correcting technical variation [46]. It is based on calculating normalization factors (NFs) for each treatment group and for each possible reference gene combination. The closer the averages of the 1/NF per treatment groups are to 1.0, the less biological variation is carried and the more accurate the expression levels of GOI (genes of interest) can be calculated. There are two indices that can be employed to rank the combinations of genes, *cv_inter_* (coefficient of variation of the 1/NFs averaged per condition), and cumulative deviation over all conditions from 1.0 of the 1/NF. As suggested, *cv_inter_* may be overruled and the cumulative deviation can be used [46]. In our study “cumulative deviation from 1.0 of the 1/NF averaged per condition” was applied and the results in Table 4 and Table S3 indicate that a combination of EF1α, HIS and RP should be used in leaf, a combination of 60S, RP, eIF-5A and GAPDH (first combination of at least three reference genes) can be used in stem, and a combination of Actin, EF1α, GAPDH, RP, and UBQ should be used in root. It is worth mentioning that GrayNorm can evaluate all possible combinations of the ten reference genes by one operation while the other three algorithms can not. It is also noteworthy that the Δ*C*_t_ method, Normfinder and RankAggreg yield in all tissues the same outcome.

**Table 4:** Best combination of reference genes based on Δ*C*_t_, Normfinder, geNorm, RankAggreg (the top-ranked reference genes), or GrayNorm, in different tissues, and given that at least three reference genes should be used for normalisation.

Algorithms	Different Tissues		
	Leaf	Stem	Root
Δ *C*_t_	*RP + HIS + eIF-5A*	*UBQ + HIS + eIF-5A*	*UBQ + eIF-5A + GAPDH*
NormFinder	*RP + HIS + eIF-5A*	*UBQ + HIS + eIF-5A*	*UBQ + eIF-5A + GAPDH*
geNorm	*60S + UBQ + eIF-5A*	*eIF-5A + UBQ + 60S + RP + HIS + TUB*	*GAPDH + GII*α *+ UBQ*
RankAggreg	*RP + HIS + eIF-5A*	*UBQ + HIS + eIF-5A*	*UBQ + eIF-5A + GAPDH*
GrayNorm	*EF1*α *+ HIS + RP*	*60S + RP + eIF-5A + GAPDH*	*Actin + EF1*α *+ GAPDH + RP + UBQ*

### 2.4. Validation of the Stability of Selected P. euphratica Reference Genes via Differential Gene Expression Analysis of Nine Putatively Salt Responsive Genes

**Figure 6:**
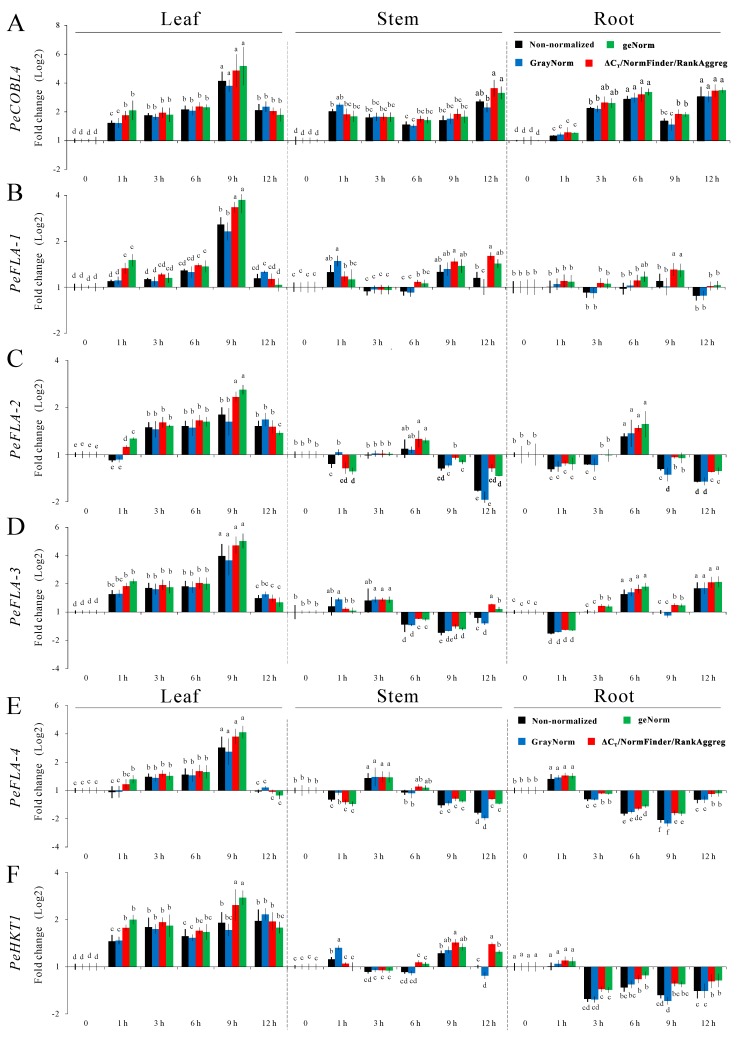

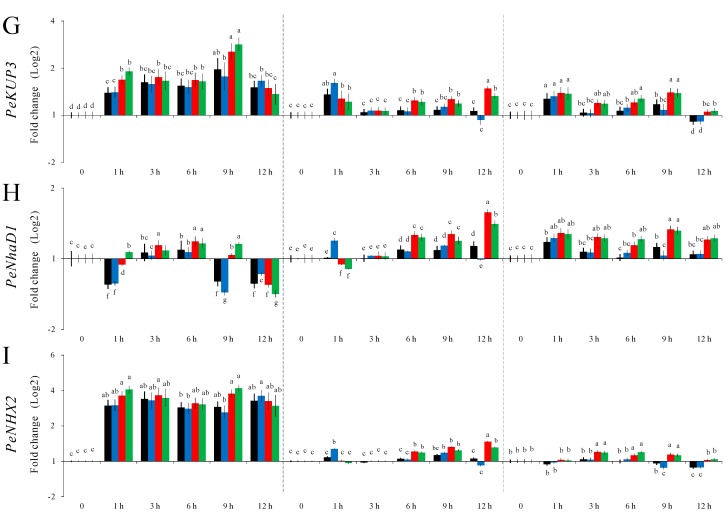
Relative expression levels of nine *P. euphratica* functional genes. Expression analysis of nine genes in response to different stages of salt stress in different tissues, based on the reference genes displayed in Table 4 recommended by different algorithms. The expression levels were log2 transformed for easily comparing the up- or down- regulation, and the “1” in *Y*-axis and “0” in *X*-axis which show “1” fold change means unchanged. **A** to **I** represent nine different genes. Error bars indicate standard errors (*n* = 3, Bars ± SE). Columns labeled with letters “a,b,c…” indicate significant differences at *p* < 0.05 between different expression levels.

The relative expression levels of nine *P. euphratica* functional genes, *PeCOBL4*, *PeFLA12-1*, *PeFLA12-2*, *PeFLA12-3*, *PeFLA12-4*, *PeHKT1*, *PeKUP3*, *PeNhaD1* and *PeNHX2*, in different tissues were analyzed by RT-qPCR. Non-normalized expression levels were compared with normalized expression levels using the reference genes selected by the various algorithms as displayed in Table 4. The non-normalized data change by using the normalization factor based on expression of the selected reference genes comprised in a normalization factor (NF). Various NFs have various effects on gene of interest expression. The smaller the difference between the normalized and non-normalized data, the more accurate the results are [46]. Figure 6 shows the induction or repression of genes by salt stress, some genes were initially induced then repressed. However, the maximum values and expression fold changes differed significantly when normalization was performed using different combinations of reference genes as proposed by the different algorithms. For example, it is known that the expression patterns of the FLA genes vary quite consistently according to the plant tissues being assessed. The mRNAs encoded by these four genes were previously found to be highly expressed in *P. euphratica* under salt stress [32]. As expected, steady-state mRNA levels for the FLA genes were induced by salt stress significantly (Figure 6). When the relative expression levels of nine functional genes were normalized with the combination recommended by GrayNorm, the fold changes were smaller comparing with Δ*C*_t_, geNorm, NormFinder or RankAggreg, and the values were closer to the non-normalized data. This indicates that GrayNorm, at least in this experiment, suggests the most optimal combination of reference genes giving the highest possible accuracy.

## 3. Discussion

Although powerful techniques, including microarrays and high-throughput measurements, have been developed to detect gene expression levels, RT-qPCR is commonly used in many laboratories. Reliable RT-qPCR data depends on suitable reference genes, which must have highly stable expression under different experimental conditions. The validation of suitable reference genes is crucial, and has been performed previously in plants [6,12,13,18,19,20]. However, in woody plants the identification of reference genes is limited to maritime pine [47], conifers [48], *Eucalyptus* [49], *Jatropha curcas* [50] and two species of *Populus*, namely, *P. trichocarpa* × *Populus deltoids* [51] and *Populus* × *euramericana* [40]. To facilitate the genetic improvement of woody plants for growth in saline soils, we performed reference gene evaluation in the salt tolerant tree *P. euphratica* and found that none of the reference genes showed perfectly stable expression (Figure 2 and Figure S4). For example, *Actin* had 3.22 cycles of variation in stem; if a target gene is normalized with *Actin* or a 0.3 cycle variation reference gene, the final relative quantification will have a 2^3.22−0.3^ = 7.57-fold discrepancy. This may fundamentally change the target gene expression profile. Thus, candidate genes showing high-level variation should be avoided as endogenous controls. More importantly, in our pre-experiments *18S* did not have significant variation, and it is often used as a candidate reference gene, such as in two *Populus* species for reference gene validation [40,51]. However, since the expression level of *18S* was too high compared with other genes, the difference value was close to ten cycles, resulting in a 1024-fold discrepancy. To the best of our knowledge, most functional genes’ expression levels are less than that of *18S*, and one of the principles in selecting suitable reference genes is that the expression level of the target and reference genes should be similar. For this reason, *18S* is rarely used as a reference gene, so we omitted it in our study and did not take it as an appropriate candidate reference gene. To further analyze the expression stability of candidate genes, several algorithms, including geNorm [11], NormFinder [43], BestKeeper [10], Δ*C*_t_ [42], qBasePlus [52], RefFinder [53], GenEx [54], GrayNorm [46], single-factor analysis of variance, and linear regression analysis [51] have been applied. Among these algorithms, geNorm, and NormFinder have the greatest impact, and the latter one is superior. NormFinder and geNorm are free of charge. Meanwhile, geNorm has been integrated into the qBasePlus and GenEx tools, both of which are powerful RT-qPCR data analysis tools.

For our data analysis, we chose the Δ*C*_t_ algorithm, the two most popular tools and a new algorithm, GrayNorm, to evaluate reference gene stability in *P. euphratica*. The Δ*C*_t_ algorithm is a primary and relatively simple approach to rank gene stability based on the SD of pair-wise differences in *C*_t_ values. This uses only *C*_q_ values for calculation, while the other three tools introduce both *C*_q_ and efficiency values. In practice, PCR efficiency is an important factor that fluctuates along with coexisting substances originating from the RNA extract and cDNA synthesis, lengths and base sequences of the primers or amplification targets, quality of the Taq enzyme, and performance of the instrument. Although these factors are not considered in the Δ*C*_t_ method, the simplicity of the algorithm makes it advantageous to use. The Δ*C*_t_ method can be used for the initial screening of reference genes from a small number of candidates since it requires manual calculation, while the other three methods can perform automated calculations.

When determining the stability of the ten reference genes, the five methods mentioned above yielded different ranking patterns. Each method has its own algorithm, some of which are *C*_q_-based (Δ*C*_t_) while others are quantity-based (NormFinder, geNorm and GrayNorm). In addition, even methods with the same base algorithm showed discrepancies; for example, when transforming *C*_q_ values to quantities to obtain input, NormFinder distributes the three *C*_q_ value repeats to three groups, while geNorm uses the average *C*_q_ values of the repeats, and GrayNorm provides all the gene combinations at one time. Meanwhile, because in all RT-qPCR analysis, GOI data are divided by *NF* values during normalization, the algorithm of GrayNorm based on 1/*NF* is more visualized. The closer the average 1/*NF* per sample group to 1.0, the more stable of the reference gene [46]. More importantly, GrayNorm displays all the gene combinations at one time from which the optimal combination can be easily found, so if only one algorithm is required, GrayNorm is a fine choice for RT-qPCR data analysis.

In previous studies on *Populus* reference gene evaluation, *P. trichocarpa* × *Populus* deltoids [51] and *Populus × euramericana* [40], both experimental conditions are in different development stages, such as overwintered terminal vegetative buds, shoot tips including unexpanded leaves and terminal vegetative buds [51], or adventitious rooting of *Populus* hardwood cuttings [40], neither of them is involved in abiotic stress. In our study, we focused on the extraordinary characteristic of *P. euphratica* on salt stress resistance. The appropriate reference genes identified in our study were different with those in above mentioned *Populus*. *UBQ*, *ACT2* and *18S* were the best three candidate reference genes in *P. trichocarpa × Populus deltoids*, *EF1*α and *18S* were the optimal two ones in *Populus × euramericana*. Meanwhile, *18S* was the optimal reference gene in potato [55], however, in our study, *18S* was omitted because of its too-high expression level. *ACT2* (Actin) and *EF1*α were middle-ranked, and only *UBQ* was top-ranked in both *P. trichocarpa × Populus deltoids* and *P. euphratica*.

Moreover, *UBQ* and *eIF-5A* were two of the best choices for use in all tissues in our study, basically corresponding to the other species, such as rice [56], soybean [18], *Oryza sativa* [57], lettuce [58] and *Arabidopsis* [59]. This may suggest that reference genes validated in *P. euphratica* have commonality with other species, and these genes are conservative. Of even greater interest, *HIS* can be considered as a novel reference gene appropriate for RT-qPCR in *P. euphratica*. However, *Actin*, widely used as endogenous control during plant development, should be avoided in *P. euphratica* under salt stress.

In *P. euphratica* DNA microarray, *GII*α have a constant expression in all experiments [60], and this indicates that it can be regarded as an ideal reference gene. However, in our study, *GII*α ranks middle-level in all tissues. Its best ranks are fourth in both root and total, behind the widely used reference gene, *UBQ* and *eIF-5A*. Because the DNA microarray was focused on water deficit stress while our research conditions are salt stress, this indicates that in different experiment treatments, there are different optimal reference genes. As water deficit leads to osmotic stress while salt condition leads to both osmotic and ion stress [61], maybe *GII*α is involved in *P. euphratica* response to ion stress, and this may have an influence on its expression stability. For *GAPDH*, it ranks seventhly in twenty-one candidate reference genes in *Arabidopsis* (*GAPDH*, AT1G13440) during development [13], and shows middle-ranks in *Eucalyptus* [49], berry [62], and tomato [63] during different environment conditions, even in human and mouse cells [64], and the validation results were similar with ours. 

Most of the nine genes’ expression levels were salt-induced as seen in Figure 6, indicating that they may participate in a salt-related mechanism in *P. euphratica*. The salt-resistance contribution may be somewhat reflected by the transcript levels in plants. *PeNHX2*, an NHX-type Na^+^/H^+^ exchanger gene, reported to play a role in mediating sodium tolerance in *P. euphratica* [65,66], showed a much higher level (fold change) than the other eight genes in leaf, and this indicated that *PeNHX2* might have a principle role in maintaining ion balance under salt conditions. The results in Figure 6 indicate that using different combinations of reference genes identified from various algorithms can influence target gene relative expression levels, and may lead to statistical significance. More importantly our results indicate that a combination of multiple reference genes recommended by GrayNorm algorithm, as shown in Table 4, should be used instead of a single reference gene. Furthermore these identified functional genes can be applied in future research in understanding of *Populus* salt-response signaling.

## 4. Experimental Section

### 4.1. Plant Materials and Salt Stress Treatment

In this study the RT-qPCR experimental steps were performed according to the protocols [67] and MIQE guidelines [68]. Seedlings of *P. euphratica* were grown in the Xinjiang Uyghur Autonomous Region of China, where they are native, after which they were transferred to an open greenhouse at Beijing Forestry University in late March the following year. They were then transplanted in individual pots containing loam soil. Each pot contained three to five individuals. The potted plants were well-irrigated according to the evaporative demand, and were grown at a natural temperature, humidity level, and photoperiod in the field for three months prior to the beginning of hydroponic culture (Figure S1). In mid-July, uniformly developed plants were washed free of soil and transferred to individual pots containing 5 L of one-quarter strength Hoagland’s nutrient solution in a closed greenhouse. The temperature in the greenhouse was 20–25 °C with a 16 h photoperiod (6 AM–10 PM) and 150 μmol·m^−2^·s^−1^ of photosynthetically active radiation. The nutrient solutions were renewed every 24 h and the plants were raised for 20–25 days under hydroponic conditions until they showed long white primary roots. The plants were then exposed to 350 mM NaCl for 12 h. The required amounts of NaCl were added to Hoagland’s nutrient solution, and plants grown hydroponically without NaCl were used as controls. Stress physiology parameters were measured at 0, 1, 3, 6, 9 and 12 h after stress treatment. The net photosynthetic rate, stomatal conductance, intercellular CO_2_ concentration, and transpiration rate were measured using the Li-6400 Photosynthesis System (Li-Cor Biosciences, Lincoln, NE, USA). Mature leaves from the same position, epidermis on stems, and primary roots were collected at 0, 1, 3, 6, 9 and 12 h after stress treatment. All samples were examined with three biological replicates and were immediately frozen in liquid nitrogen and stored at −80 °C.

As the factors which affect the gene expressions are salt stress and diurnal changes, we are looking at a combination of both of them. Based on the principle of single factor under material treatments, we strongly recommend the other researchers to actually include time-point controls in their own experiments, and analyze reference genes and genes of interest in treated and control samples at each time-point [46].

### 4.2. RNA Extraction, Quality Control, and cDNA Preparation

Stored samples were ground in liquid nitrogen to powder with a mortar and pestle. Total RNA was extracted using the cetyl trimethyl ammonium bromide (CTAB) method for plants [69]. Genomic DNA was eliminated by treating the RNA samples with DNase in the last step of the extraction process and with gDNase in the first step of the reverse transcription process. The quantity and quality of RNA were assessed with a NanoDrop2000 spectrophotometer (Thermo Fisher Scientific, Waltham, MA, USA) by detecting the OD_260_/OD_280_ and OD_260_/OD_230_ ratios, respectively. The RNA samples were also examined using 1% agarose gel electrophoresis for 15 min, and the integrities were examined using an Agilent 2100 Bioanalyzer (Agilent Technologies, Santa Clara, CA, USA) according to the manufacturer’s instructions. Equal amounts of total RNA (1.8 μg) were reverse-transcribed using a TIANGEN FastQuant RT Kit (with gDNase) (Qiagen, Hilden, Germany) according to the manufacturer’s protocol; 20 μL of purified cDNA samples were diluted approximately 1:10 to the same concentration in RNase-free water.

### 4.3. Selection of P. euphratica Candidate Reference Genes and Functional Genes, and Primer Design

*GII*α has already been used as a reference gene in the gene expression analysis of *P. euphratica* under water deficit for four weeks [60], and was added in this study. From the transcriptomics of salt-responses [30,31,32,38,70,71] and the whole sequenced genome data of *P. euphratica* [26], half of the candidate reference genes can be found, and all the nine functional genes were chosen. Moreover, our results were compared with other forest species such as Eucalyptus [72] on the reference gene validation conclusions [49]. In general, half of the candidate reference genes were widely used in model plants like *Arabidopsis* and rice, and the others were come from above mentioned transcriptomics of *P. euphratica*. Ten potential housekeeping genes were selected as candidate reference genes based on the previous research [40,41]. Nine functional genes, *PeCOBL4*, *PeFLA12-1*, *PeFLA12-2*, *PeFLA12-3* and *PeFLA12-4* were selected from transcriptional data of *P. euphratica* [32]. *PeHKT1*, *PeKUP3*, *PeNhaD1* and *PeNHX2* were selected from the genome data of *P. euphratica* [26]. To compare the stability of the reference genes between different tissues or just in leaves, primer sequences from previous studies were used [41]. The annealing temperature of the primers was 60 ± 3 °C and the length was 18–30 bp. The amplicon was between 80 and 200 bp while the GC content ranged from 40% to 60%.

### 4.4. RT-qPCR Reaction Conditions

RT-qPCR was conducted in triplicate in 96-well plates with an ABI StepOne^Plus^ instrument (ABI, Carlsbad, CA, USA). A TIAGEN SuperReal PreMix Kit (Qiagen, Hilden, Germany) with SYBR Green detection chemistry was used. The reaction volume was 20 μL containing 1 μL of diluted cDNA (corresponding to 9 ng of total RNA), 0.3 μM each primer, 10 μL of 2× PreMix Plus, and 2 μL of ROX Reference Dye. The cycling parameters were 95 °C for 15 min, followed by 45 cycles of 20 s at 95 °C and 60 s at 60 °C. Melting curves for each amplicon were then performed by heating the amplicon from 40 to 100 °C and reading at each temperature to verify the specificity of each amplification reaction. Negative controls without template were performed at the same time. The PCR products were also examined on a 2% agarose gel for 30 min to determine the amplicon size and specificity of the amplification reaction.

### 4.5. Determination of Reference Gene Expression Stability Using ΔC_t_, NormFinder, geNorm and GrayNorm

A flowchart was constructed to explain the data analysis strategy. Four calculation methods, including Δ*C*_t_ [42] and three publicly available tools, NormFinder [43], geNorm [11] and GrayNorm [46] were used for expression stability validation of the ten genes in *P. euphratica* tissues, referred to previous study on *Coffea* species. [73]. The Δ*C*_t_ approach was employed by comparing the relative expression of “pairs of genes” within each sample. Δ*C*_t_ uses the raw *C*_q_ values as input. The mean standard deviation (SD) values of the Δ*C*_t_ values (mSD) were used to rank the expression stability of the ten genes. NormFinder software (http://moma.dk/normfinder-software) is a Visual Basic Application (VBA) based on Excel for ranking the expression stability of reference genes. The lowest calculated value indicates the most stable expression. geNorm (http://medgen.ugent.be/~jvdesomp/genorm/), another VBA applet, was also used to rank the candidate reference genes based on the average expression stability values index, M. *C*_q_ values were transformed to obtain input, and the most stable gene with the lowest *M* value was ranked on the right. geNorm software can also be used to calculate another important index, the pairwise variation (PV), to determine the optimal number of control genes for use in normalization [11]. Download links, summaries, or detailed directions for all these methods can also be found at the website (http://www.gene-quantification.de/download.html) or for GrayNorm (https://github.com/gjbex/GrayNorm#graynorm).

### 4.6. Gene Stability Rankings Merged Using RankAggreg

The RankAggreg v0.4-3 (http://cran.r-project.org/web/packages/RankAggreg/) package of R program v3.0.1 (http://www.r-project.org/), which provides an easy and convenient interface to handle complex rank aggregation problems [74], was used to merge the stability measurements obtained from the three methods and establish a consensus rank of reference genes. There are two different algorithms found in RankAggreg, and according to the size of the ranking list (ten), we used the Cross-Entropy Monte Carlo algorithm (CE) to visually present the rank aggregation using a line chart. The rankings from four terms, mSD, stability, coefficient of variance (*CV*), and *M*, were used as input. The most stable two gene orders evaluated using geNorm were distinguished based on the initial *M* value when calculating the normalization factor (*NF*) value. The other bioinformatics and statistical analysis softwares used in this study including SPSS v19.0 (http://www-01.ibm.com/software/analytics/spss/), SigmaPlot v12.5 (http://www.sigmaplot.com/) and qBasePlus v2.5 (http://www.biogazelle.com/).

### 4.7. Expression Analysis of Nine P. euphratica Functional Genes

To explore the salt responses in the 18 *P. euphratica* samples and illustrate the suitability of those identified reference genes, we analyzed the relative expression levels of nine *P. euphratica* functional genes; namely, *PeCOBL4*, *PeFLA12-1*, *PeFLA12-2*, *PeFLA12-3*, *PeFLA12-4*, *PeHKT1*, *PeKUP3*, *PeNhaD1* and *PeNHX2*, in different tissues using the optimal or worst reference genes.

*PeCOBL4* gene which encodes an extracellular glycosyl-phosphatidylinositol-anchored protein, showing a distinctly different expression level in *Populus* *×* *canescens* (*P* *×* *c*) and *P. euphratica* (*P. eu*) under salt stress, and in *P. euphratica* the expression level is high [32]. *PeFLA12-1*, *PeFLA12-2*, *PeFLA12-3* and *PeFLA12-4* displayed similar expression patterns with *PeCOBL4* in two *Populus* species [32]. In *P. euphratica* the Na^+^/K^+^ transporter *PeHKT1* is similarly expanded as tandem duplicated copies in *T. salsuginea* [75] and *T. parvula* [76], and it responds to salt stress rapidly [26]. *PeKUP3* and *PeNhaD1* were significantly up-regulated under salt stress when compared with salt-sensitive poplar, *P. tomentosa*, and both of them are involved in ion transport and homeostasis [26,65]. The transcript levels of *PeNHX2* were up-regulated in roots after NaCl treatment for 6 h, and heterologous expression of *PeNHX2* in yeast mutant strain R100 can improve salt tolerance [66].

The single best or worst reference gene was based on RankAggreg results, while the combination was recommended by both geNorm and RankAggreg. *C*_q_ values and amplification efficiency values were processed using the qBasePlus software [52]. For calculating the relative expression levels of the nine functional genes based on a combination of multiple reference gene, such as the six top-ranked reference genes, *HIS* + *eIF-5A* + *UBQ* + *GII*α + *GAPDH* + *EF1*α. Firstly, we calculated out the relative quantification (RQ) of a functional gene, such as *PeCOBL4*, using *E*^Δ*C*t^ method. *E* was the PCR efficiency of *PeCOBL4* gene, 1.887, as shown in Table 1. Δ*C*_t_ means the *C*_q_ values of stressed plants (1, 3, 6, 9 and 12 h in the stress stage) take away the *C*_q_ values of non-stressed plants (0 in the stress stage). Secondly, a “Normalization factor” (NF) needed to be calculated, the “NF” was calculated by geNorm or qBasePlus (geNorm is incorporated into qBasePlus now) software after log-transformed reference genes’ *C*_q_ values were input, and the first column are reference genes’ names and the first row are samples’ names. Then dividing the “RQ” value of *PeCOBL4* by the “*NF*” value, we get final relative expression levels. This is also the working principle of qBasePlus and geNorm according to the manuals.

## 5. Conclusions

Overall, to identify suitable reference genes, we propose using the Δ*C*_t_ method to examine gene expression stability before subsequent evaluation by NormFinder, geNorm, GrayNorm, or other software programs. Our results provide an important reference gene selection guide when performing gene expression analysis in different tissues of *P. euphratica*.

## Supplementary Materials

Supplementary materials can be found at http://www.mdpi.com/1422-0067/16/09/20468/s1.
