# Supplementary material for: Evaluation of Appropriate Reference Genes for Reverse Transcription-Quantitative PCR Studies in Different Tissues of a Desert Poplar via Comparision of Different Algorithms
Source: Int J Mol Sci. 2015 Aug 28;16(9):20468–91. doi: 10.3390/ijms160920468 (PMC4613214; doi:10.3390/ijms160920468)
Supplement: Supplementary file 1 [file ijms-16-20468-s001.zip › ijms-85003-Supplementary Information/ijms-85003-Supplementary Information.pdf]

Supplementary Information

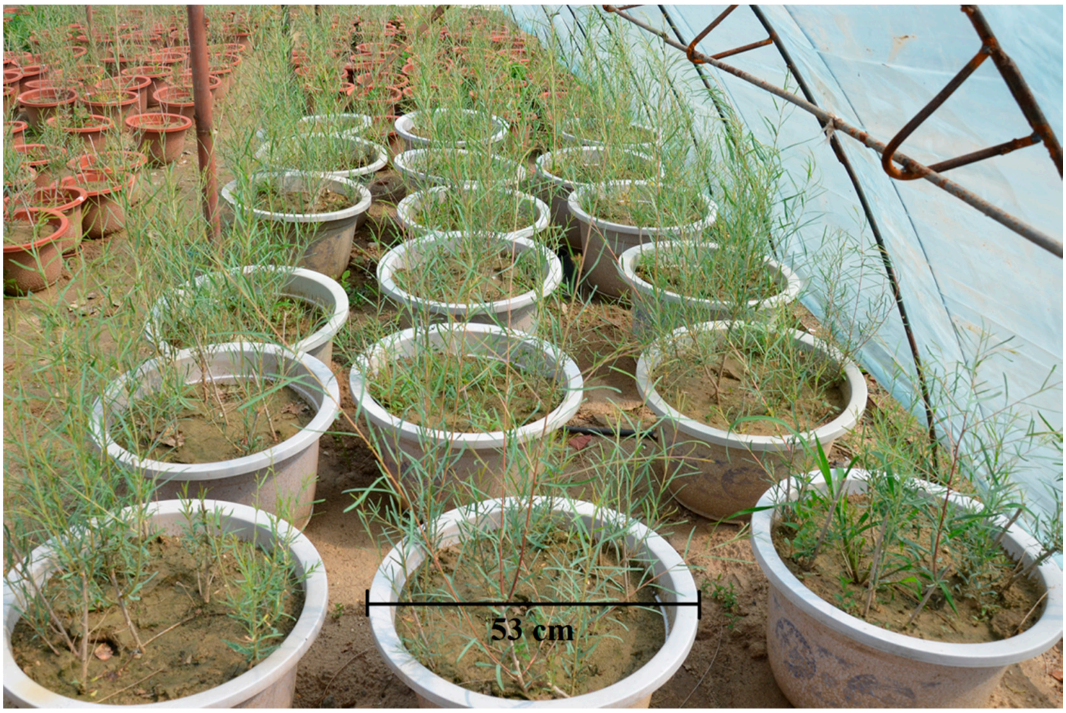

Figure S1. The *P. euphratica* saplings used in this study.

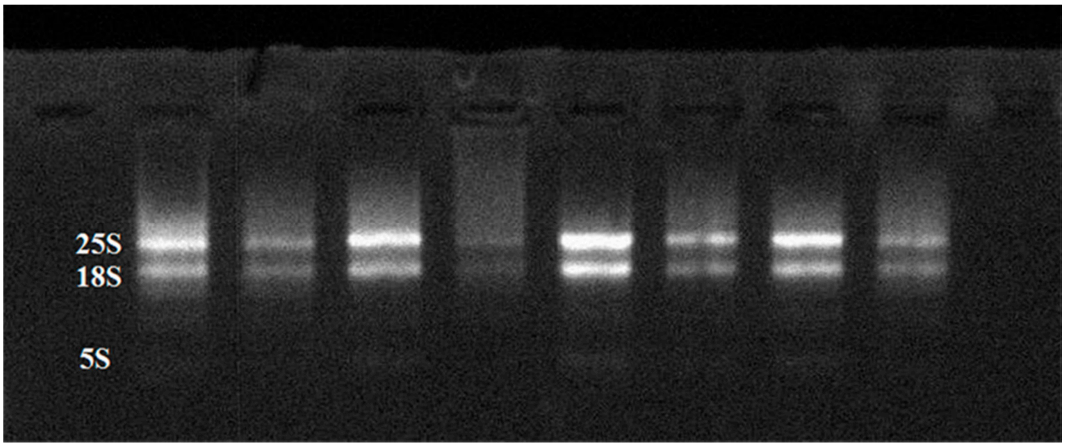

Figure S2. Total RNA quality detected using 2% agarose gel electrophoresis.

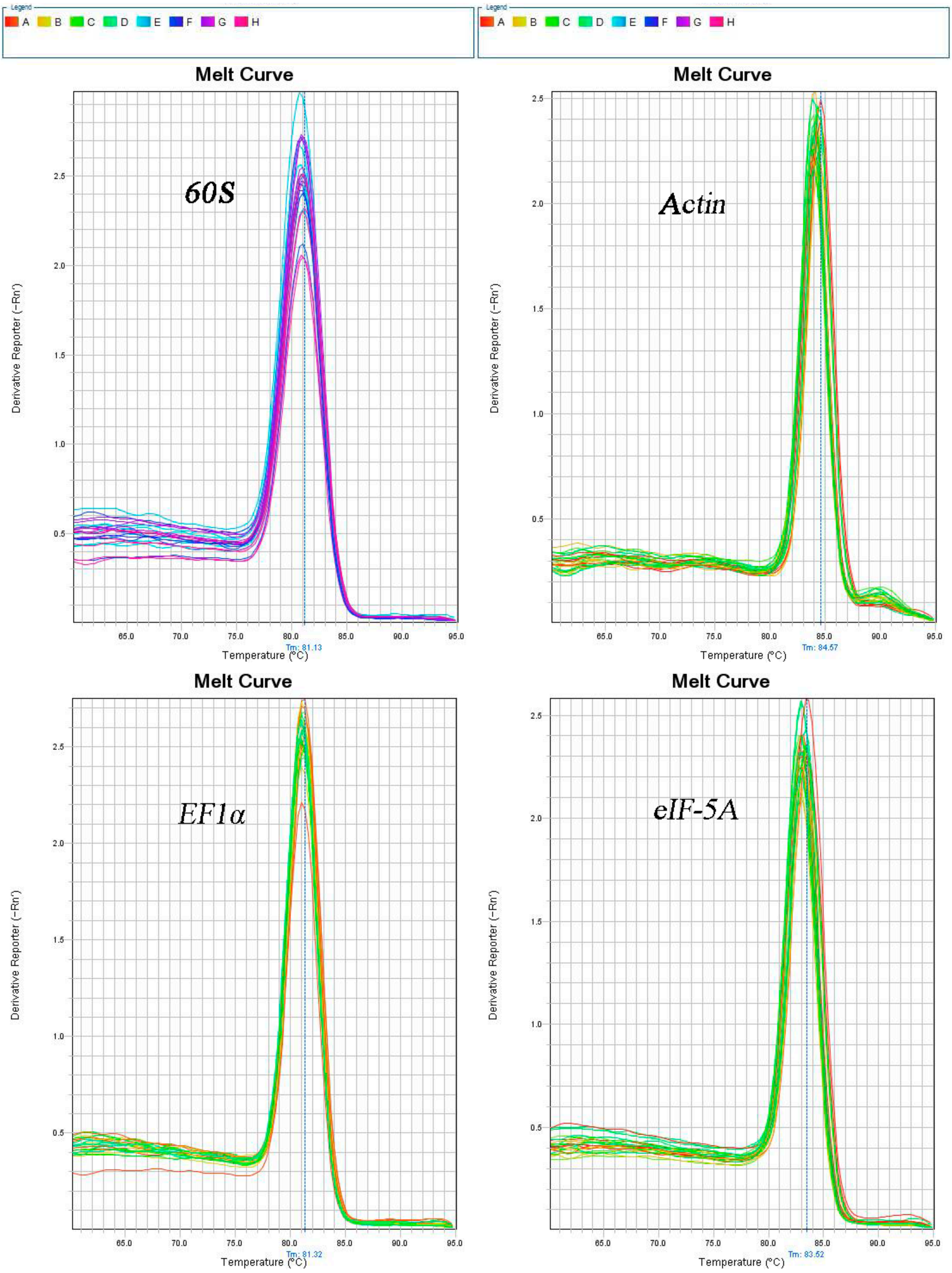

Figure 3. *Cont.*

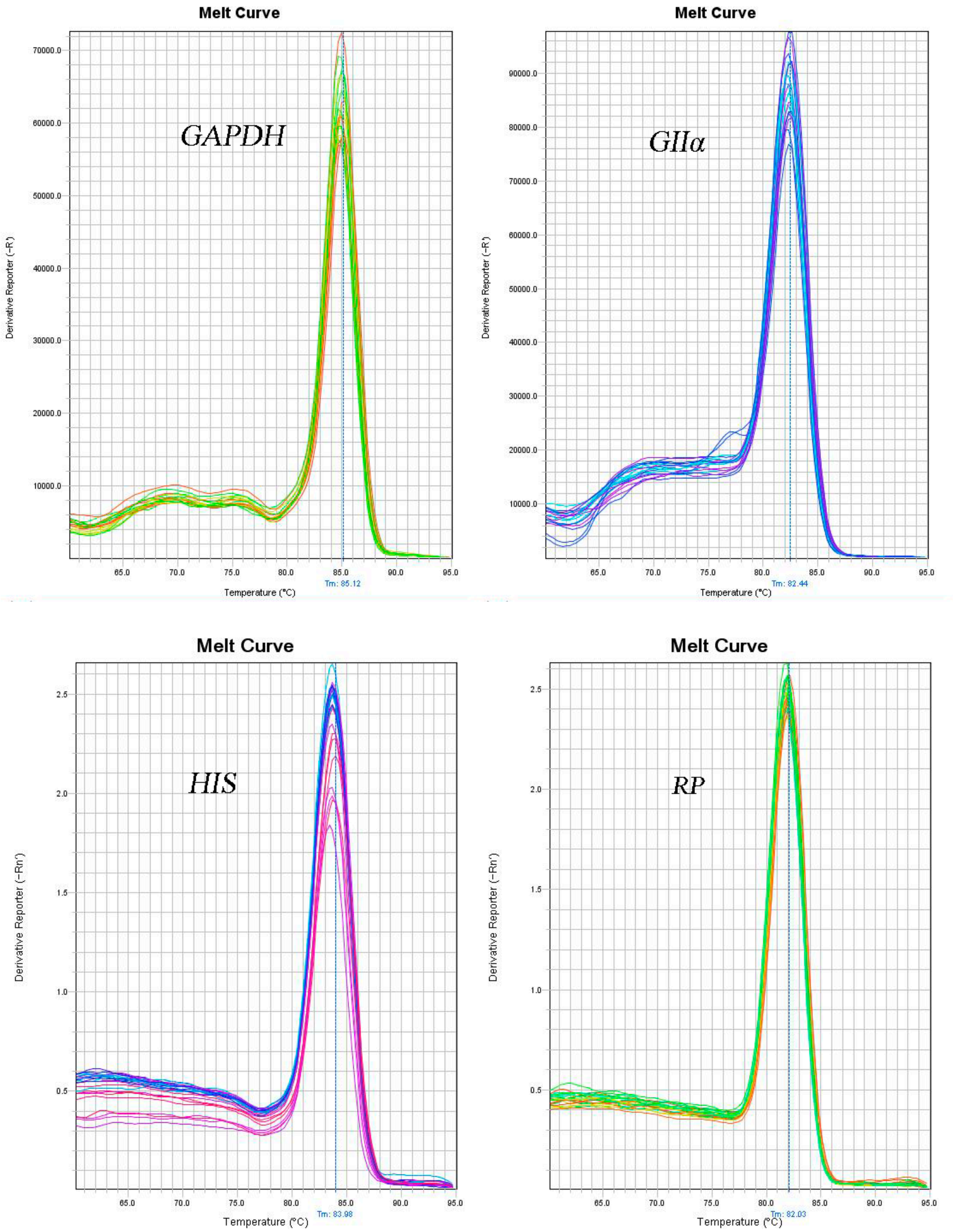

Figure 3. *Cont.*

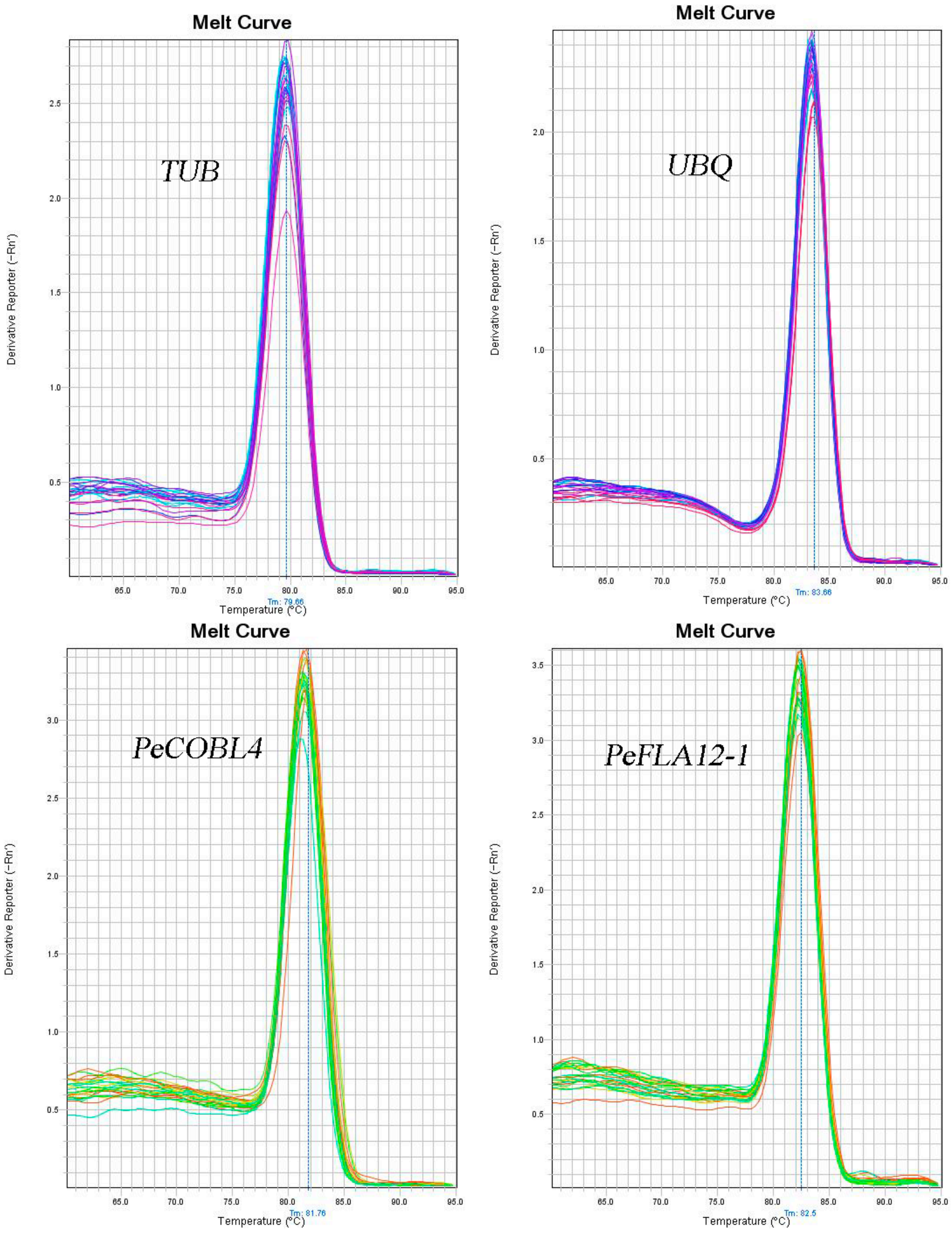

Figure 3. *Cont.*

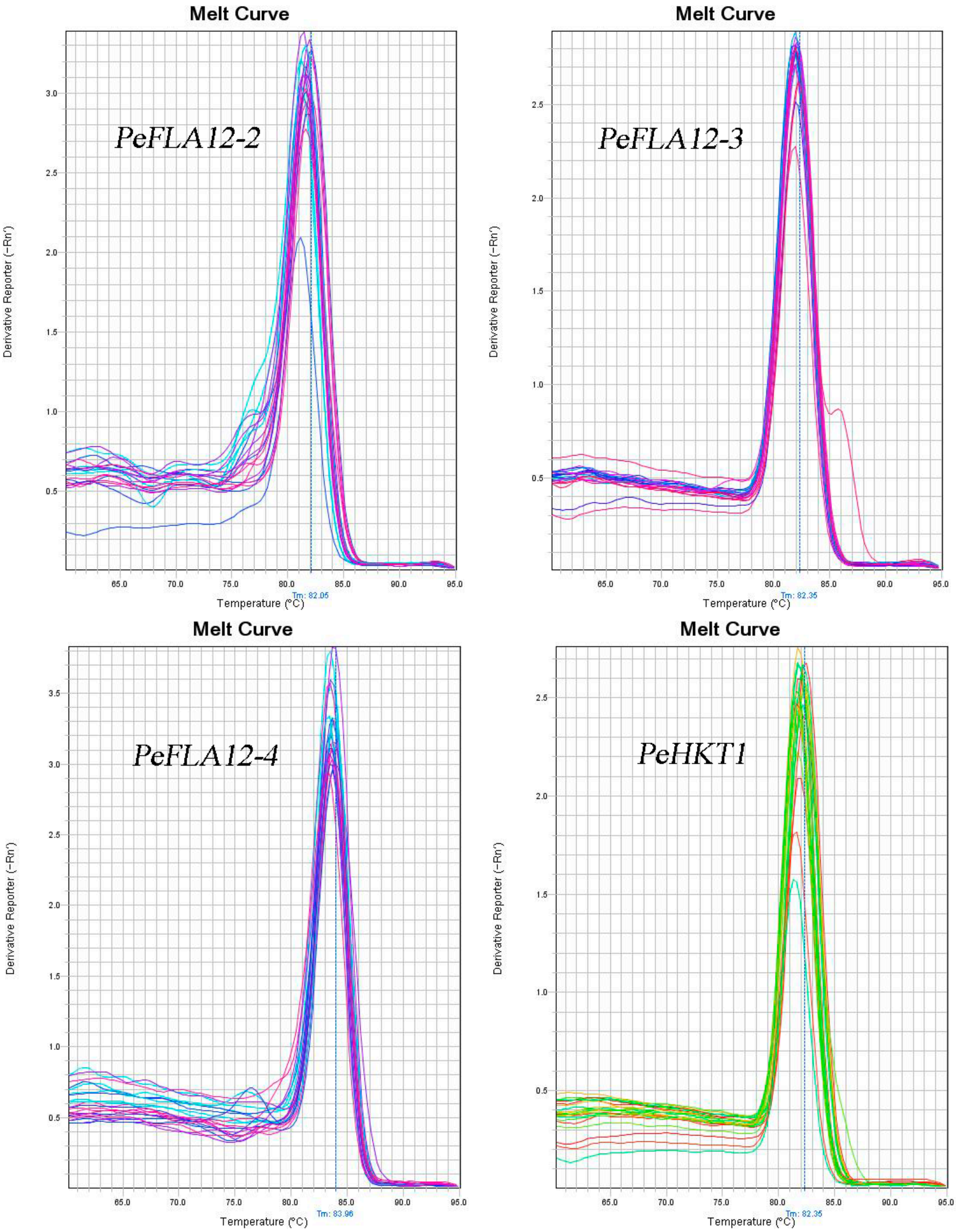

Figure 3. *Cont.*

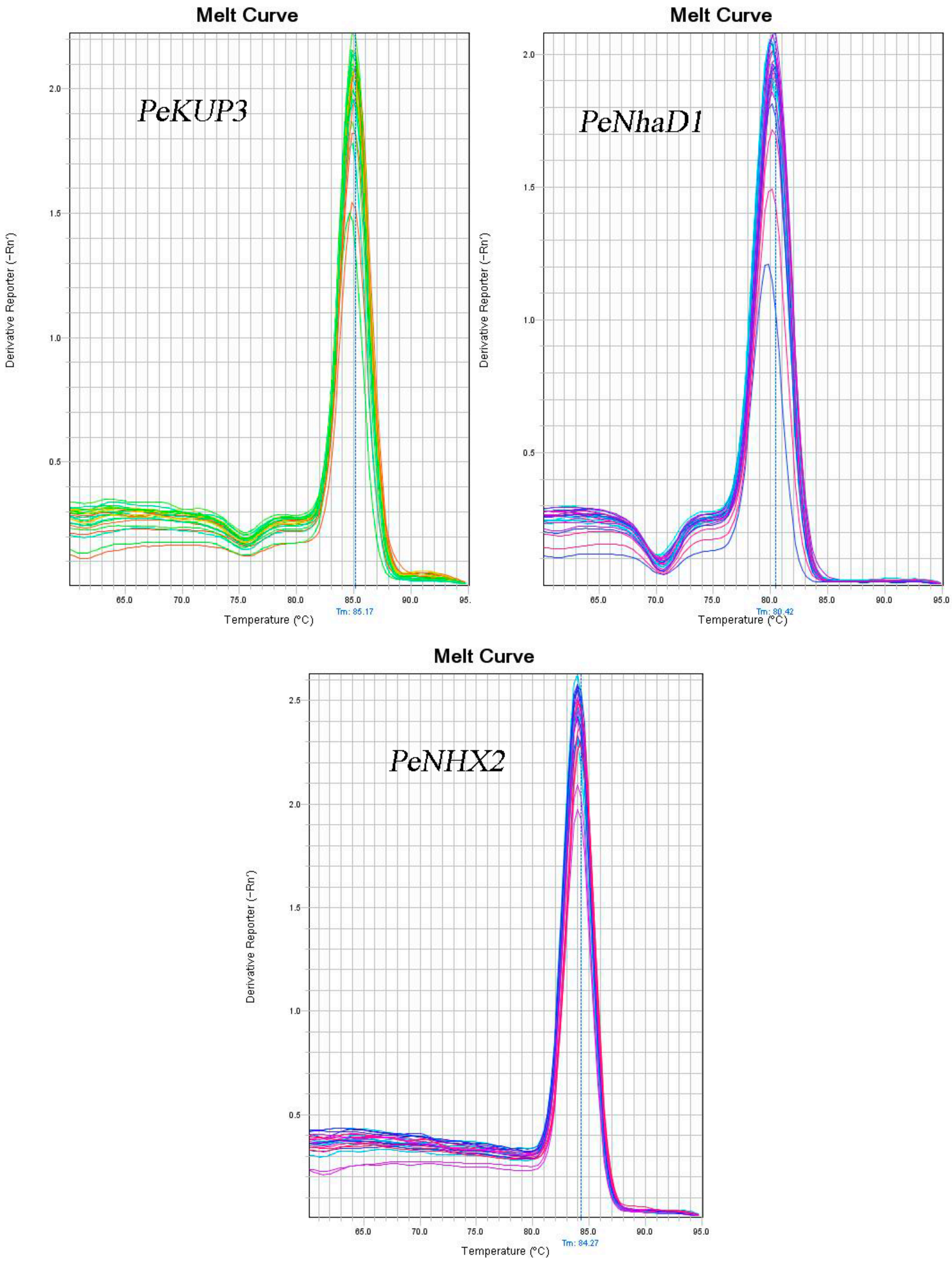

Figure S3. Melting curves of the ten reference genes and nine functional genes for qRT-PCR amplification specificity detection.

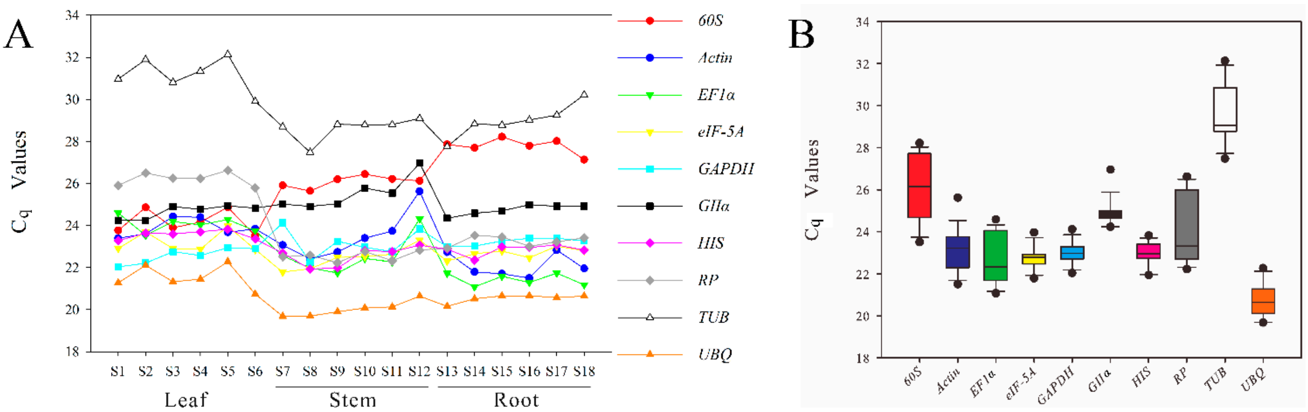

Figure S4. Expression levels of the ten candidate reference genes across 18 experimental *P. euphratica* samples. (**A**) Mean C_q values of the ten candidate reference genes with three replicates; and (**B**) Box-whisker plot showing the C_q variation among all tested samples. Dots indicate outliers.

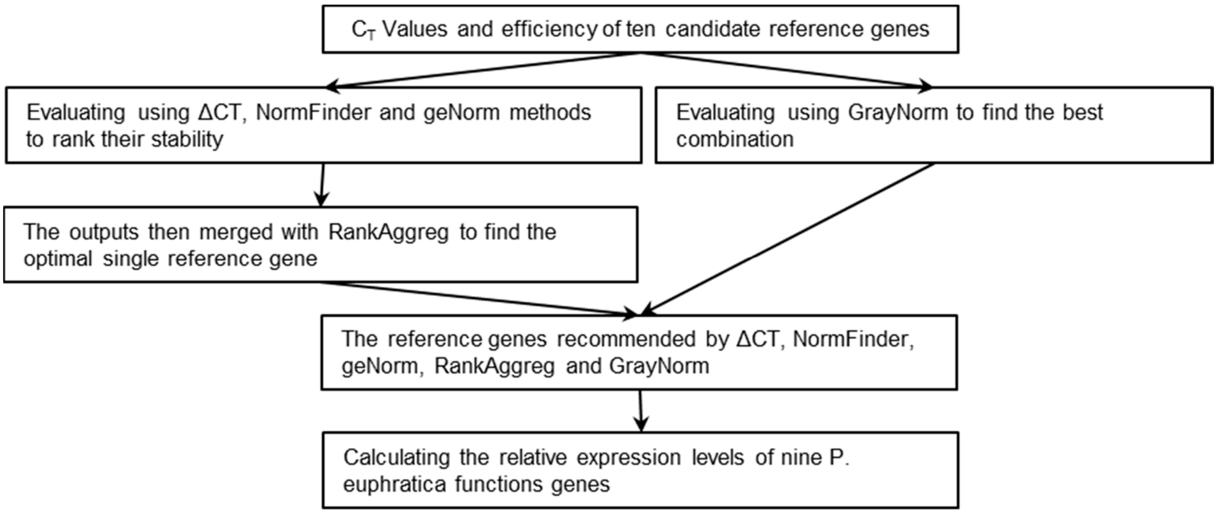

Figure S5. A flowchart for data analysis strategy.

Table S1. MIQE précis for this study using template table (Bustin *et al.*, 2010). "√" represents that we performed the experiments.

Sample/Template	Details	Checklist
Source	Mature leaves, stem epidermis and healthy roots were collected from salt-stressed, one-year-old *P. euphratica* saplings. Please see Figure 1 in manuscript and Figure S1.	√
Method of preservation	All fresh tissue samples wrapped with aluminium foil paper were immediately frozen in liquid nitrogen and stored at −80 °C.	√
Storage time (if appropriate)	The frozen mature leaves, stem epidermis and healthy roots were stored at −80 °C from July to August, 2014 (two months). Total RNA was extracted and reverse transcribed to cDNA, and both of the RNA and cDNA were stored at −80 °C along with the experiment processes (Four months, from September to December, 2014).	√
Handling	The frozen samples were ground in liquid nitrogen to a fine powder for three times with a pestle and a mortar.	√
Extraction method	The cetyltrimethyl ammonium bromide (CTAB) method for trees were used for total RNA extraction. Modification: dithiothreitol (DTT) was used in place of 2% β-mercaptoethanol; precipitate at −20 °C for 4 h in place of 2 h.	√
RNA: DNA-free	(1) For RNA-specific precipitation (few genomic DNA), 1/4 volume of 10 mol/L lithium chloride (LiCl) was added to the supernatant and mixed in step 3 of the protocol; (2) Potentially contaminating genomic DNA was eliminated by treatment with DNase I (TAKARA #D2270A, Kusatsu, Japan) in the last step of the protocol; (3) Reverse transcription kit (with gDNase) was used (TIANGEN FastQuant RT Kit, with gDNase, 100rxn, Cat#KR106-02, Lot#N3009, Qiagen, Düsseldorf, Germany); (4) Most of the primers are Intron-spanning; (5) We compared the C_t values obtained with and without reverse transcription (RT) for the samples, and all the difference of the C_t values were between 14 to 22.	√
Concentration	NanoDrop2000 spectrophotometer (Thermo, West Palm Beach, FL, USA) was used for assessing the RNA and cDNA concentration. In stem and root samples, the RNA concentration varied from 349.4 μg/μL (OD_{260}/OD_{280} was 2.11, OD_{260}/OD_{230} was 2.13) to 492.2 μg/μL (OD_{260}/OD_{280} was 2.07, OD_{260}/OD_{230} was 1.71). In leaf samples, the RNA concentration varied from 542.7 μg/μL (OD_{260}/OD_{280} was 2.10, OD_{260}/OD_{230} was 1.71) to 667.4 μg/μL (OD_{260}/OD_{280} was 2.09, OD_{260}/OD_{230} was 1.56).	√
RNA: Integrity	Agilent 2100 Bioanalyzer (Agilent Technologies, Santa Clara, CA, USA), mostly for high-throughput sequencing RNA quality control, was used for RNA integrity detection of one third of the representative RNA samples according to the manufacturer's instructions.	√
Inhibition-free	Dilution: 1.8 μg of total RNA was reverse-transcribed in 20 μL volume. Then the cDNA was diluted 1:10 with nuclease-free water.	
**Assay Optimisation/Validation**		
Accession number	The detailed accession numbers of the nineteen genes (ten candidate reference genes and nine functional genes) can be seen in Table 1 of the manuscript or "Table S2" in this file.	√
Amplicon details	Amplicon size can be seen in "Table S2" in this file.	√
Primer sequence	The primer sequence of each target can be seen in Table 1 of the manuscript or "Table S2" in this file.	√
Probe sequence *	n/a	

Table S1. *Cont.*

Sample/Template	Details	Checklist
In silico	The Phytozome v9.1 GI (http://www.phytozome.net/, *Populus trichocarpa*) was used for gene BLAST. For Primer-BLAST the NCBI website (http://www.ncbi.nlm.nih.gov/tools/primer-blast/index.cgi?LINK_LOC=BlastHome) and Primer Premier 6 software were both used. The RTPrimerDB (http://medgen.ugent.be/rtprimerdb/) was also used as a contrast. For Primer specificity detection, Figure S3 (Melting curves) in Supplementary Materials are available.	√
Empirical	2 OD of primers were synthesized by Sangon Biotech (Shanghai, China), and the primers were diluted to 20 μmol/L, 0.3 μL was used and the final concentation in the reaction was 0.3 μmol/L for each primer. For annealing temperature please see "Table S2" in this file.	√
Priming conditions	TIANGEN FastQuant RT Kit (with gDNase) (#KR106-02, Qiagen, Germany) was used, and FQ-RT Primer Mix, RT Enzyme Mix, and 10× Fast RT Buffer were used in the second step of the reverse transcription reaction (42 °C 15 min, 95 °C 3 min).	√
PCR efficiency	PCR efficiency and slopes of the calibration curves can be seen in "Table S2" in this file. The content of cDNA was diluted 4-, 8-, 16-, 32- and 64-fold.	√
Linear dynamic range	In our study, it was not multiplexing amplification, only one target was amplified in each tube because only one peak of melting curver was observed and only one expected band exist in agarose gel electrophoresis, so this step is not necessary.	√
Limits of detection	In our RT-qPCR experimental conditions, 1 μL of cDNA (20 μL diluted 1:10 with nuclease-free water, correspond to 9 ng RNA) was used in each tube, when diluted 1:10, 1:100, 1:1000; 1:10,000 for calucating PCR efficiency, we can also get the accurate amplification curve.	
Intra-assay variation	The PCR measurements and results indicated that ten candidate refernence genes are of high copy numbers.	
**RT/PCR**		
Protocols	For reverse transcription: 1.8 μg RNA, 2 μL 5× gDNA Buffer, add RNase-Free ddH$_2$O up to 10 μL volume for 42 °C 3 min reaction, then add 2 μL 10× Fast RT Buffer, 1 μL RT Enzyme Mix, 2 μL RT Primer Mix, and 5 μL RNase-Free ddH$_2$O up to 20 μL volume for 42 °C 15 min, 95 °C 3 min reaction. Then the cDNA was diluted 1:10 with nuclease-free ddH$_2$O water. For RT-qPCR:The reaction volume was 20 μL containing 1 μL of diluted cDNA (corresponding to 9 ng of total RNA), 0.3 μM each primer, 10 μL of 2× PreMix Plus, and 2 μL of ROX Reference Dye. The cycling parameters were 95 °C for 15 min, followed by 45 cycles of 20 s at 95 °C and 60 s at 60 °C. Melting curves for each amplicon were then performed by heating the amplicon from 40–100 °C and reading at each temperature to verify the specificity of each amplification reaction.	√
Reagents	For reverse transcription: TIANGEN FastQuant RT Kit, with gDNase, 100rxn, Cat#KR106-02, Lot#N3009, Qiagen, Düsseldorf, Germany). For qPCR: TIANGEN SuperReal qPCR PreMix(SYBR Green) and ROX (#FP204, Qiagen, Düsseldorf, Germany).	√
Duplicate RT	1.8 μg of total RNA was used in every sample, two replicates of the reverse transcription step were also applied. For standard deviation of the C_t values, please see Figure 2 of the manuscript.	√
NTC	NTCs were included and gave no amplification. Melt curves can be seen in Figure S4.	√
NAC	The no amplification controls (NAC) provide valuable information when using probes, as they help monitor any probe degradation. In this SYBR method, we performed the NAC and no fluorescence signals were detected.	√
Positive control	All the C_t values of the ten candidate reference genes except *TUB* (relatively low copy numbers) are concentrated between 20 and 30, they are very close to those of the nine functional genes.	

Table S1. *Cont.*

Sample/Template	Details	Checklist
**Data Analysis**		
Specialist software	RT-qPCR was conducted in triplicate in 96-well plates with an ABI StepOnePlus instrument (ABI, Carlsbad, CA, USA). For calculating the relative expression levels of the nine functional genes based on a combination of multiple reference gene, such as the five top-ranked reference genes, Actin + EF1α + GAPDH + RP + UBQ, recommended by GrayNorm in root. Firstly, we calculated out the relative quantification (RQ) of a functional gene, such as PeCOBL4, using $E^{(-\Delta CT)}$ method. E was the PCR efficiency of PeCOBL4 gene, 1.887, as shown in Table 1. ΔC_t means the C_t values of stressed plants (1, 3, 6, 9 and 12 h in the stress stage) take away the C_t values of non-stressed plants (0 in the stress stage). Secondly, a "Normalization factor" (*NF*) need to be calculated, the "*NF*" was calculated by geNorm or qBasePlus (geNorm is incorporated into qBasePlus now) software after log-transformed reference genes' C_t values were input, and the first column are reference genes' names and the first row are samples' names. Then dividing the "RQ" value of PeCOBL4 by the "*NF*" value and we get final relative expression levels. This is also the working principle of qBasePlus and geNorm according to the manuals.	√
Statistical justification	Three biological replicates were performed for each gene, and statistical test was also performed.	√
Transparent, validated normalisation	To make the readers easily understand our work, we provided a flowchart in "Supporting Material", and displayed all the download links of the tools, we also provided our calculating processes in " Experimental Section", Section 4.7. Multiple validated reference genes were used for normalization and non-normalized data were also presented.	√

Table S2. Detailed information of the ten candidate reference genes and nine functional genes, and primer sequences can be seen in Table 1 in the manuscript.

Gene Name	Tentative (Annotation)	Phytozome v9.1 GI (*P. trichocarpa*)	*Arabidopsis* (Ortholog Locus)	*E* Values	GC Content (Forward Primers, Reverse Primers)	Annealing (Temperature, F/R, °C)	Amplicon (Length, bp)	Dilution (Curve Slope)	PCR (Efficiency)
60S	60S ribosomal RNA	Potri.007G093700	AT5G65220	$1.10 \times E^{-51}$	44.0%, 48.0%	59.1/59.6	177	−3.391	1.972
Actin	Actin family 3	Potri.006G192700	AT3G12110	0	50.0%, 48.1%	59.8/59.9	160	−3.259	2.027
EF1α	Elongation factor-1 α	Potri.006G130900	AT1G07940	0	50.0%, 56.5%	59.6/59.4	100	−3.599	1.896
eIF-5A	Eukaryotic initiation factor 5A	Potri.018G107300	AT1G13950	$1.80 \times E^{-100}$	60.0%, 44.0%	58.9/59.0	118	−3.409	1.965
GAPDH	Glyceraldehyde-3-phosphate dehydrogenase	Potri.010G055400	AT3G04120	0	54.2%, 48.0%	59.9/59.4	138	−3.576	1.904
GIIα	Glucosidase II α-subunit	DQ388455.1	AT5G63840	0	50.0%, 57.9%	54.3/52.8	65	−3.301	2.009
HIS	Histone superfamily protein H3	Potri.005G072300	AT4G40040	$4.70 \times E^{-95}$	52.2%, 59.1%	57.5/59.9	111	−3.401	1.968
RP	Ribosomal L27e protein family	Potri.001G342500	AT3G22230	$9.40 \times E^{-69}$	52.2%, 47.8%	58.3/58.8	136	−3.488	1.935
TUB	Tubulin β chain	Potri.003G126800	AT5G23860	0	54.2%, 50.0%	59.2/60.1	138	−3.547	1.914
UBQ	Ubiquitin family 6	Potri.014G115100	AT2G47110	$7.40 \times E^{-87}$	48.0%, 59.1%	60.1/60.2	139	−3.109	2.097
PeCOBL4	COBRA-like extracellular glycosyl-phosphatidyl	Potri.004G117200	AT5G15630	0	44.0%, 57.9%	57.9/59.7	110	−3.626	1.887
PeFLA12-1	FASCICLIN-like arabinogalactan-protein 12–1	Potri.009G012100	AT5G60490	$5.60 \times E^{-50}$	41.4%, 60.0%	59.6/58.1	89	−3.464	1.944
PeFLA12-2	FASCICLIN-like arabinogalactan-protein 12–2	Potri.009G012200	AT5G60490	$5.80 \times E^{-50}$	46.2%, 48.0%	60.5/58.4	92	−3.250	2.031
PeFLA12-3	FASCICLIN-like arabinogalactan-protein 12–3	Potri.012G015000	AT5G60490	$1.20 \times E^{-54}$	40.7%, 54.2%	60.2/60.6	121	−3.156	2.074
PeFLA12-4	FASCICLIN-like arabinogalactan-protein 12–4	Potri.004G210600	AT5G60490	$3.60 \times E^{-57}$	48.0%, 46.2%	60.7/60.2	174	−3.210	2.049
PeHKT1	High-affinity K^+ transporter 1	Potri.018G132200	AT4G10310	$1.30 \times E^{-137}$	46.2%, 41.4%	60.1/58.8	168	−3.199	2.054
PeKUP3	K^+ uptake transporter 3	Potri.014G144900	AT3G02050	0	52.2%, 54.2%	58.9/59.9	177	−3.376	1.978
PeNhaD1	NhaD-type Na^+/H^+ antiporter 1	JX981308	AT3G19490	0	48.0%, 52.0%	60.0/60.2	126	−3.437	1.954
PeNHX2	Na^+/H^+ exchanger 2	Potri.014G134900	AT3G05030	0	52.0%, 56.5%	59.7/58.5	194	−3.182	2.062
